# Machine learning-ready remote sensing data for Maya archaeology

**DOI:** 10.1038/s41597-023-02455-x

**Published:** 2023-08-23

**Authors:** Žiga Kokalj, Sašo Džeroski, Ivan Šprajc, Jasmina Štajdohar, Andrej Draksler, Maja Somrak

**Affiliations:** 1 Research Centre of the Slovenian Academy of Sciences and Arts (ZRC SAZU), Novi trg 2, 1000 Ljubljana, Slovenia; 2 Information and Communication Technologies, Jožef Stefan International Postgraduate School, Jamova cesta 39, 1000 Ljubljana, Slovenia; 3 Jožef Stefan Institute, Jamova cesta 39, 1000 Ljubljana, Slovenia

**Keywords:** Archaeology, Geography

## Abstract

In our study, we set out to collect a multimodal annotated dataset for remote sensing of Maya archaeology, that is suitable for deep learning. The dataset covers the area around Chactún, one of the largest ancient Maya urban centres in the central Yucatán Peninsula. The dataset includes five types of data records: raster visualisations and canopy height model from airborne laser scanning (ALS) data, Sentinel-1 and Sentinel-2 satellite data, and manual data annotations. The manual annotations (used as binary masks) represent three different types of ancient Maya structures (class labels: buildings, platforms, and aguadas – artificial reservoirs) within the study area, their exact locations, and boundaries. The dataset is ready for use with machine learning, including convolutional neural networks (CNNs) for object recognition, object localization (detection), and semantic segmentation. We would like to provide this dataset to help more research teams develop their own computer vision models for investigations of Maya archaeology or improve existing ones.

## Background & Summary

Airborne laser scanning (ALS) surveys have proved crucial for advancement of knowledge in archaeological “site” distribution, particularly in the forested regions of the ancient Maya^1–3^, as they have greatly accelerated and expanded traditional archaeological landscape surveys. The research use of ALS in landscape archaeology typically involves the identification, localisation, recording and investigation of natural and cultural features for a variety of, usually interrelated, contexts, including but not limited to the mapping and analysis of settlement, urbanism, agricultural production and water management^4–11^.

Archaeologists typically inspect ALS data in the form of raster visualisations, which enhance the perception of surface features^12–14^. Human visual analysis and digitisation is time-consuming and the examination of hundreds of square kilometres can take months, depending on the level of detail, number of structures, and the recording method. Despite the lack of large-scale, high-resolution, publicly available ALS datasets of the ancient Maya region, dispersed private and public funding has made it possible to conduct not only site-specific landscape studies over a few square kilometres e.g.^7,15–18^, but also large-scale studies over several hundred or even several thousand square kilometres e.g.^3,5,11,19–23^. The volume of data makes it difficult to annotate entire datasets, especially if not only the locations of objects, but also their shape is to be indicated. The subjectivity of human visual inspection and digitisation and the variability between human interpreters is also an issue^24^. There is therefore a pressing need to employ computer vision methods that can find archaeological objects and delineate their boundaries automatically^25,26^. Among the various machine learning approaches, deep convolutional neural networks (CNNs) are the current state-of-the-art for computer vision, but they usually require a large number of already labelled samples^27^ for training. This makes labelled datasets crucial for developing and testing the methods.

In one of our own previous studies, we have already demonstrated that CNNs can classify ancient Maya archaeological objects from DEM visualisations, achieving up to 95% accuracy^28^. However, classification models do not have the potential to replace manual inspection and labelling, for which semantic segmentation is required. Semantic segmentation is readily applied in remote sensing ^a review is given by^^27^, but even more so in medical imaging, where CNNs often outperform experts^29–34^.

The original intention for collecting the ALS data in the area around Chactún, one of the largest ancient Maya urban centres known so far in the central lowlands of the Yucatan Peninsula, was to better understand the water management, agriculture, settlement dynamics and socio-political organisation of the ancient Maya living in this area^11,35^.

We generated a labelled dataset that can be used for the analysis of ancient Maya archaeology comprising more than 10.000 objects, divided into three different classes; buildings, platforms, aguadas (artificial water basins). We used polygonised outlines of objects to create binary raster masks. The associated multimodal dataset contains data from three remote sensing sources:

- 0.5 m resolution ALS data visualisations^28^ (sky view factor, positive openness, slope),
- 1.0 m resolution ALS canopy height model,
- 10 m resolution Sentinel-1 Short Aperture Radar (SAR) satellite data (annual average Sigma0), and
- 10, 20 and 60 m resolution Sentinel-2 optical satellite data (12 bands + s2cloudless cloud mask, 17 dates).

**Fig. 1:**
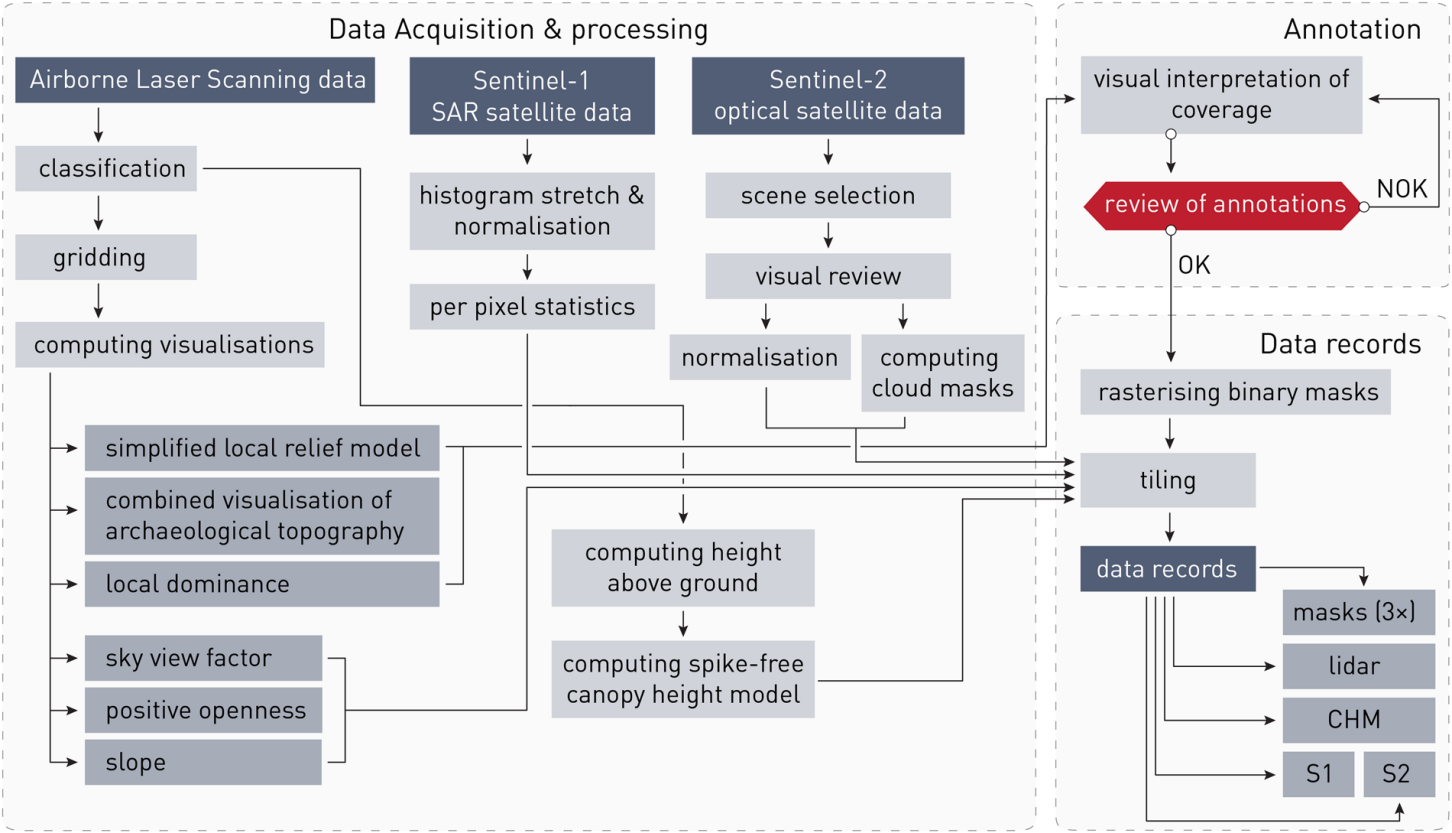
An overview of the experimental workflow used to generate and analyse the data.

Sentinel-1 and 2 Earth observation missions are part of the European Union Copernicus Programme. An overview of the experimental workflow used to generate and analyse the data is presented in Fig. 1.

The application of CNN methods in archaeological prospection has just begun to gain momentum e.g.^28,36–41^; currently, however, there are only a few CNN-based semantic segmentation studies conducted with ALS data^26,42–46^, and even fewer instance segmentation models published in this particular field^47^. Easily accessible, archaeologically labelled datasets suitable for machine learning are therefore extremely rare. We believe that sharing a large labelled dataset that allows semantic segmentation, because it is based on polygonised objects rather than centroids, points, or simple bounding boxes, has great reuse value. This dataset is also unique because it is multimodal and, to date, the only one in the Maya region. Such a rich dataset allows related research groups to develop or improve their own segmentation models. This has already led to improvements in recognition rates, as the dataset was used in the Discovering the mysteries of the Maya machine learning competition^48,49^. The teams that took part in this machine learning challenge achieved a segmentation performance of more than 0.83 for the intersection over union (IoU, also known as the Jaccard index) when learning from ALS data. However, most teams did not include satellite data in their final model. Deep learning from ALS visualisations alone produced better results with much less machine learning engineering effort. However, by providing a multimodal dataset for a wider reuse, we hope other teams can develop new models based on architectures that can better harness the information in the satellite data.

## Methods

### Site description

**Fig. 2:**
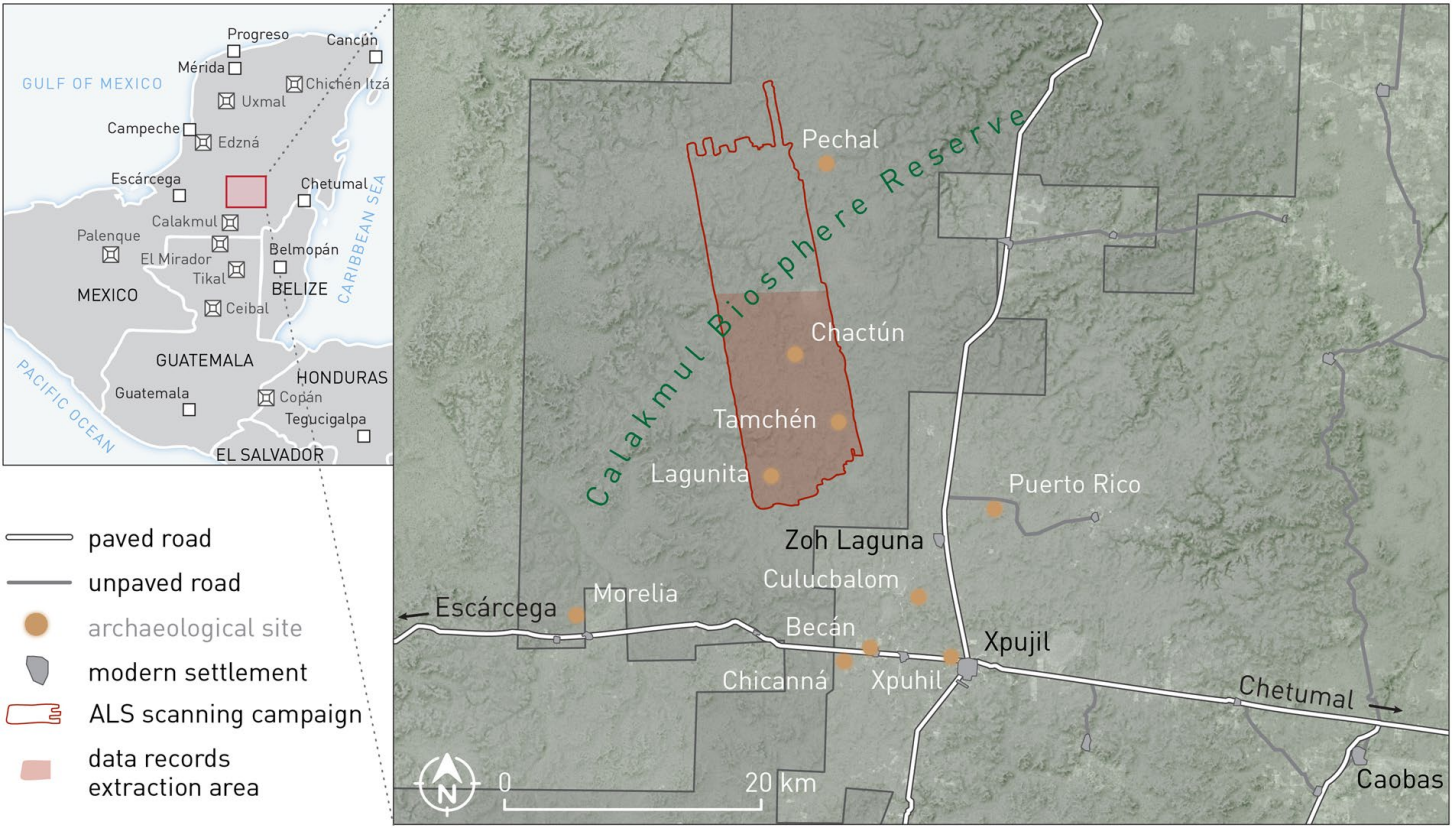
Location of the study area with delineated area of the airborne laser scanning mission. Data records were extracted from its southern part (coloured in red) adapted from^28^.

The area around Chactún (Fig. 2) is karstic and therefore lacks perennial water and permanent water streams. Low hills typically rise up to 30 m above the surrounding seasonal wetlands (*bajos*). The climate in the Maya lowlands is tropical and isothermal^50,51^ and within the elevated interior region rainfall is highly seasonal and spatially variable. Typically, 90% of precipitation occurs during the rainy season^9^. The entire study area is covered by natural, unmanaged, tropical semi-deciduous forest and bushes, rarely exceeding 20 m in height. The forest can be classified as primary forest, where there has been no agricultural or grazing activity for a millennium. Before the establishment of the Calakmul Biosphere Reserve in 1989, selective logging for valuable timber and chicle collection were the main economic activities.

Šprajc and his team discovered the urban core of Chactún, composed of three concentrations of monumental architecture, in 2013^52^. Temple-pyramids, massive palace-like buildings and two ball courts surround its several plazas. A large rectangular water reservoir lies immediately to the west of the main groups of structures. Ceramics collected from the ground surface, the architectural characteristics and dated monuments indicate that the centre began to flourish in the Preclassic period (c. 1000 BC–250 AD), and reached its climax during the Late Classic (c. 600–1000 AD) playing an important role in the regional political hierarchy^52,53^. South of Chactún are Lagunita and Tamchén, both prominent urban centres. Numerous smaller building clusters are scattered on the hills around them^54,55^.

### Survey information

The whole dataset includes five different types of data records:

- airborne laser scanning (ALS) raster visualisations,
- ALS data derived canopy height model,
- Sentinel-1 synthetic aperture radar satellite data,
- Sentinel-2 optical satellite data, and
- data annotations.

### Airborne laser scanning data

The main part of the dataset consists of visualised airborne laser scanning data collected with the Titan system by the National Centre for Airborne Laser Mapping (NCALM) at the end (peak) of the dry season in May 2016. Mission planning, data acquisition and data processing were carried out with clear archaeological objectives in mind^56,57^. The density of the final point cloud and the quality of the derived elevation model with a 0.5 m spatial resolution (Table 1) proved to be excellent for the detection and interpretation of archaeological features with very clearly defined minute elevation differences.

**Table 1:** ALS data acquisition parameters of the region around Chactún, Calakmul Biosphere Reserve, Campeche, Mexico.

scanner type	Optech Titan
platform	fixed wing
date	between 17 and 20 May 2016
laser wavelength (3 channels) [nm]	1550 (infrared); 1064 (near-infrared); 532 (green)
swath width [m]	600
flying height [m]	800–900
overlap [%]	50
average last and only returns per m^2^ on a combined dataset (pulse density)	28.2
average classified ground returns per m^2^ on a combined dataset	12.8
spatial resolution of the final elevation model [m]	0.5

The technical quality control of the data included verification of the scanning density, the absolute horizontal accuracy (better than 20 cm), the absolute vertical accuracy (better than 15 cm), and the thematic accuracy of the produced elevation model.

**Fig. 3:**
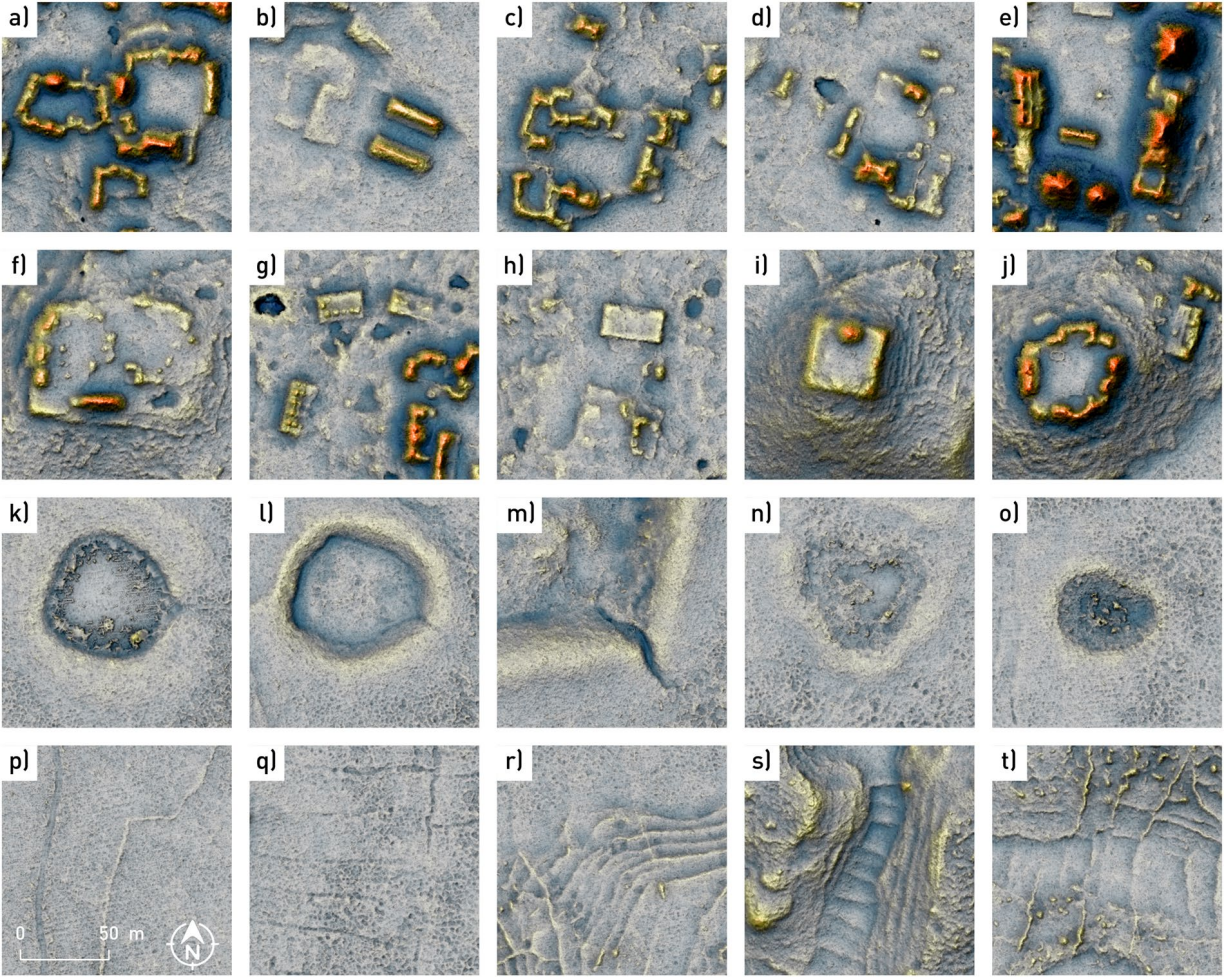
Examples of buildings (**a**–**g,i,j**), platforms (**a**–**j**), and aguadas (**k**–**o**) that have been annotated and are included in the dataset. Other man-made structures such as walls, channels, terraces, rock-piles, etc. were not annotated (**p**–**t**). All panels have the same scale and cover about a quarter of a single data record area (Fig. 5). The visualisation is for illustrative purposes only; it combines a coloured simplified local relief model with a combined visualisation for archaeological topography.

Ground points were classified using Terrascan software (version 016.013), which uses an adaptive triangular irregular networks densification algorithm^58^. The algorithm settings were optimised to remove only the vegetation cover, leaving the remains of past human activities as intact as possible (Table 2). Ground points therefore include remains of buildings, walls, terraces, mounds, *chultuns* (cisterns), *sacbeob* (raised paved roads), and drainage channels (Fig. 3). Rare areas without ground returns include aguadas with water. Many landscape features, such as ditches and low field walls, were essentially invisible in the field, due to dense vegetation, and would most likely have been missed by conventional surface mapping. As revealed by ground-truthing, the elevation model contains very few data collection and processing artefacts (commission and omission errors). Instances of omission errors are limited to smaller objects, such as altars, while commission errors mostly include larger tree trunks. In some places, parts of buildings are misshapen, for example walls being classified as vegetation because a tree is growing from a chamber on top of a pyramidal building.

**Table 2:** Ground classification processing parameters^13^.

maximum building size [m]	30
terrain angle [°]	89
iteration angle [°]	9
iteration distance [m]	1.4
reduce iteration angle edge length [m]	<5

We used raster ALS data visualisations to support the human vision interpretation of objects and as a three-band dataset for the data records. ALS raster data visualisations are computed derivatives of a digital elevation model that provide information about the landscape. They can have a purely presentational value or can relate to physical quantities^59^. In effect, they facilitate ‘reading and exploring’ the landscape in search of meaningful information. The primary visualisation was a combined visualisation for archaeological topography (VAT)^13^. It blends two combinations of four distinct visualisations: analytical hillshading, slope, positive openness and sky-view factor (SVF)^12^, computed with settings for normal and flat terrain (Table 3). The individual visualisation methods are complimentary, depicting small topographic variations in different ways, and the combined image preserves their positive characteristics. Before blending, the visualisations are normalised and have a custom histogram stretch applied. Having a single combined visualisation to consider has advantages, including better representation of structures in a wider range of terrain types, conservation of disk space, and faster display. VAT was created in the Relief Visualization Toolbox (RVT; https://github.com/EarthObservation/RVT_py). The calculation takes about half a minute per km^2^ for data at 0.5 m resolution on a normal office laptop. VAT has already been used for pedestrian surveys in a range of environments, from semi-deciduous tropical forests of Mexico to the largely open heather and scrubland of western Scotland and the karstic, rugged terrain of the Mediterranean. The visualisation does not introduce artefacts and shows small-scale features well, regardless of their orientation and shape. However, it is not completely orientation independent, as hillshading is used as the base layer. It aids human vision interpretation, but uses directional light source and is therefore not suitable for data augmentation techniques such as rotation and flipping. Therefore, only sky-view factor, positive openness, and slope, which are all direction independent, were used to create three-band (RGB) data records. These also ranked highest in our study of the performance of different visualisations and visualisation combinations for the classification task with CNNs^28^. Local dominance^12^ served as an additional aid for human vision interpretation of outer boundaries of aguadas, which are usually very faintly raised above the surrounding flat terrain.

**Table 3:** Raster visualisations used to create the combined visualisation for archaeological topography that we used to identify objects. It blends four raster visualisations computed with settings for general and flat terrain. A colour coded simplified local relief model was added to improve the visibility of structures (Fig. 3) and local dominance was used for delineating the outer edges of aguadas. Sky-view factor, positive openness, and slope, computed with settings for general terrain, are used as raster bands in the data records.

	general terrain		flat terrain		blending
**visualisation**	**settings**	**normalisation**	**settings**	**normalisation**	**order, opacity, type**
sky-view factor	5 m radius in 16 directions	linear, 0.7–1.0	10 m radius in 16 directions, excluding the first 4 m	linear, 0.9–1.0	3, 25%, multiply
positive openness	5 m radius in 16 directions	linear, 68°–93°	10 m radius in 16 directions, excluding the first 4 m	linear, 85°–93°	2, 50%, overlay
slope	inverted greyscale colour bar	linear, 0.0°–50°	inverted greyscale colour bar	linear, 0.0°–15°	1, 50%, luminosity
hillshading	35° Sun angle, 315° azimuth	linear, 0.0–1.0	15° Sun angle, 315° azimuth	linear, 0.0–1.0	0, base layer
simplified local relief model	10 m radius, custom colour bar	linear, −6 m–6 m			1, 100%, colour (overlaying Combined VAT)
local dominance	5 m–10 m radius	linear, 0.5–1.8			

In addition, we provide a canopy height model (CHM) with a resolution of 1 m, computed with a spike-free algorithm^60^ implemented in the LAStools las2dem tool (version 230330). The processing parameters are listed in Table 4. Based on visual inspection, we removed all points that are more than 30 m above the ground (or a building), as they represent noise rather than a true measurement of tree heights.

**Table 4:** Spike-free canopy height model processing parameters.

drop points below height [m]	0.2
drop points above height [m]	30
spike free (freeze constraint) [m]	3
kill (triangulation limit) [m]	5
step (resolution) [m]	1

The raw ALS data was provided by ZRC SAZU as part of a collaboration between the authors of this paper. As Chactún, Lagunita and Tamchén have only recently been (re)discovered and are remote and difficult to access, their exact location is not known to the general public. The density of ancient Maya anthropogenic structures and terrain modifications in this area is astonishing, reaching the level of mayor urban centres like Tikal, but is still almost completely unexplored archaeologically. To prevent looting, the locations of the urban cores and the numerous smaller settlement groups are restricted to researchers. The full ALS data is therefore not publicly available. However, investigators who wish to use it for a specific application should approach ZRC SAZU directly by contacting the corresponding author, describing the topic and goals of their project.

### Sentinel-1 synthetic aperture radar satellite data

The Sentinel-1 satellite constellation provides C-band Synthetic Aperture Radar (SAR) data. The first satellite, Sentinel-1A, was launched in April 2014, followed by Sentinel-2 in April 2016. The latter was decommissioned after data collection ceased due to power failure on 23 December 2021. The dual constellation had a repetition frequency of 6 days and a revisit frequency (in ascending and descending orbit) of 3 days at the equator. A single satellite has a revisit frequency of 6 days at the equator.

For this study, we used data acquired by both satellites in the Interferometric Wide (IW) swath mode, as this is the primary acquisition mode over land with the largest data archive. We used the Level-1, Ground Range Detected (GRD) product with dual polarisation (Vertical Transmit – Horizontal Receive Polarisation (VV) and Vertical Transmit – Vertical Receive Polarisation (VH)) for both ascending (ASC) and descending (DES) orbits. The backscatter coefficient used was Sigma0. The values of the backscatter coefficient were converted from linear power to decibels (dB), fitted to an interval of [−30, 5] dB, and normalised to the range [0, 1].

We used SAR data for the years 2017–2020, with 114 ascending orbit (ASC) images and 205 descending orbit (DES) images, collected from Sentinel Hub^61^. A total of 319 Sentinel-1 images were acquired over the study area, each containing data for VV and VH polarisation with 10 m spatial resolution. We calculated the following temporal statistics for each pixel: mean, median, standard deviation, coefficient of variance, and 5^th^ and 95^th^ percentiles. We calculated the statistics for each year within the observation period and for the entire period. The data were stored as a multiband raster (120 bands) in the Tagged Image File Format (TIFF) format (Fig. 5 and Table 8). All processing was done with our own code and the Python packages eolearn (version 1.4.2) and sentinelhub (version 3.9.1).

Due to the complete absence of modern anthropogenic objects and measured permanent scatterers in our study area, it was impossible to verify the positional accuracy of the SAR data. However, according to the Sentinel-1 Annual Performance Report^62^, the geolocation accuracy of the IW swath mode products without geometric corrections is −3.5 m for range and 2.1 m for azimuth. The absolute localisation error is therefore well below the mission requirements of 10 m at 95% confidence.

### Sentinel-2 optical satellite data

The Sentinel-2 optical satellite mission began with the launch of Sentinel-2A in June 2015, followed by Sentinel-2B in March 2017. Both satellites carry a single multi-spectral instrument (MSI) with 13 spectral channels in the visible/near-infrared (VNIR) and shortwave infrared spectral range (SWIR). The spatial resolution is 10 m for four bands, 20 m for six bands and 60 m for three bands (Table 5).

**Table 5:** The spatial and spectral resolution of Sentinel-2 MSI bands^66^.

band	resolution [m]	Sentinel-2A		Sentinel-2B	
		central wavelength [nm]	bandwidth [nm]	central wavelength [nm]	bandwidth [nm]
1	60	443	20	442	20
2	10	493	65	492	65
3	10	560	35	559	35
4	10	665	30	665	31
5	20	704	14	704	15
6	20	741	14	739	13
7	20	783	19	780	19
8	10	833	105	833	104
8a	20	865	21	864	21
9	60	945	19	943	20
10	60	1374	29	1377	29
11	20	1614	90	1610	94
12	20	2202	174	2186	184

**Fig. 4:**
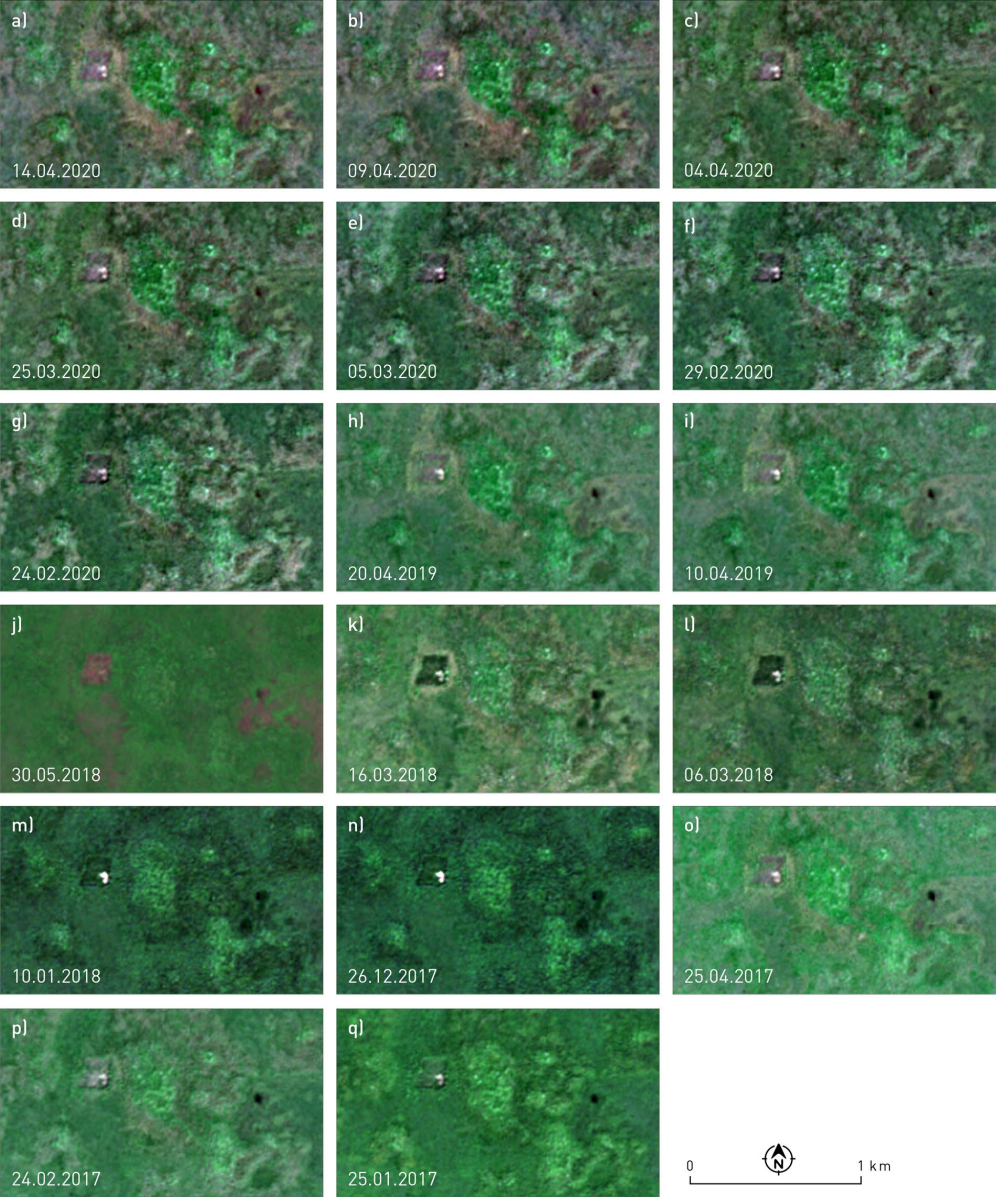
The selected Sentinel-2 acquisition dates over the study area with close-up views.

**Fig. 5:**
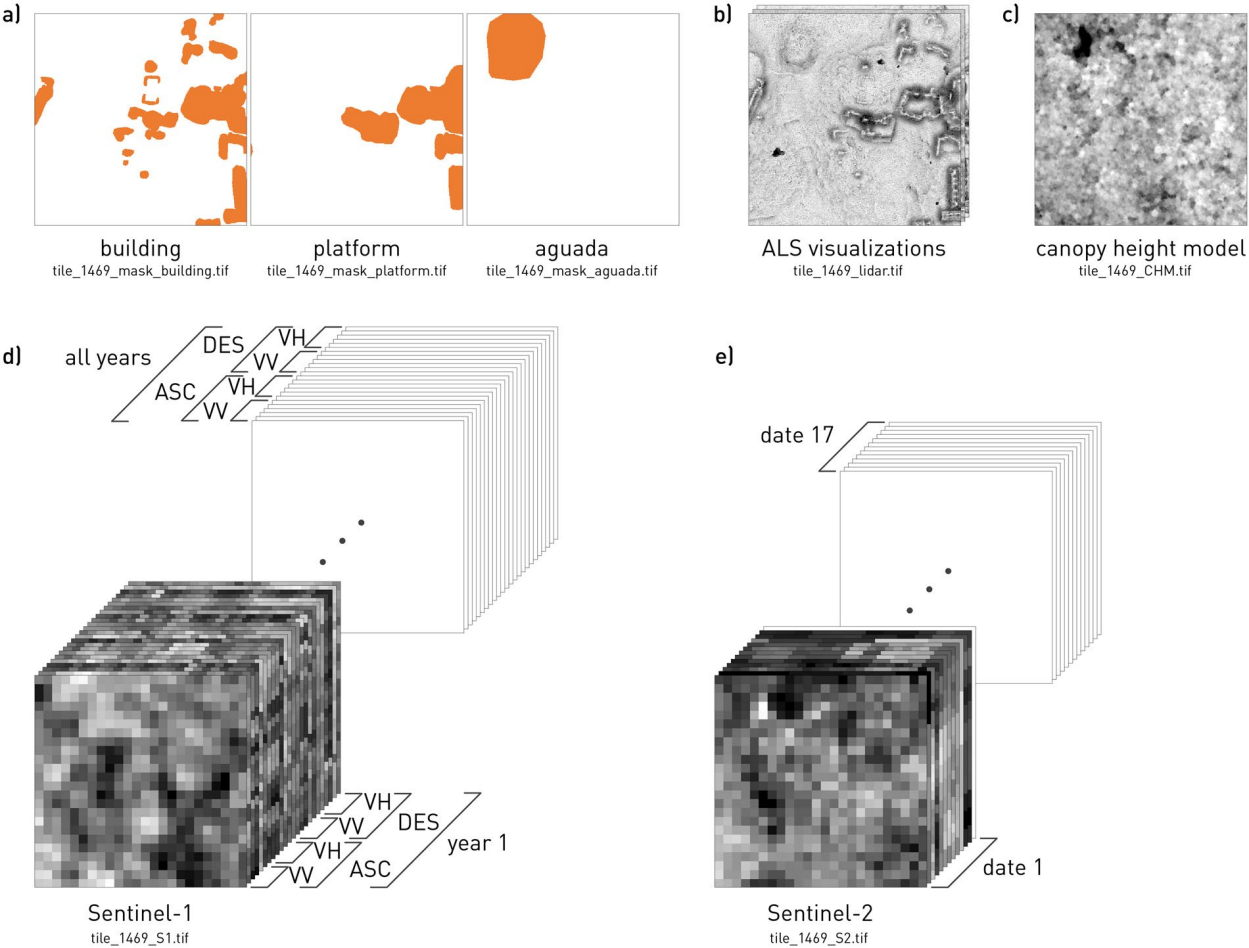
A single data record (e.g. 1469) contains (**a**) binary masks of structures, (**b**) a tile with ALS data visualisations, (**c**) a tile with a canopy height model, (**d**) a tile with annual statistics for Sentinel-1 Sigma0 backscatter coefficients, and (**e**) a tile with Sentinel-2 bands for 17 cloudless scenes.

The geographical and climatic characteristics of the study area are manifested in a high proportion of cloudy optical satellite images. Out of 658 Level-2A images acquired during 2017–2020^61^, 78 have cloud cover of less than 5%, however, small convective clouds or haze are present in most of them. We calculated a cloud mask with a 10 m resolution for each acquisition date and performed a manual visual inspection of the set to finally select the 17 images without cloud cover over the study area. We resampled all bands to 10 m resolution using the nearest neighbour resampling method. The dataset therefore comprises 12 spectral bands (excluding band 10) and a corresponding cloud mask, computed using s2cloudless^63^ (available at https://github.com/sentinel-hub/sentinel2-cloud-detector) adjusted to 10 m resolution, for 17 dates (Fig. 4) (221 bands in total), saved in the TIFF format (Fig. 5 and Table 8). We excluded spectral band 10 (also known as the cirrus band) because it does not contain surface information. It is used for atmospheric corrections and is therefore not included in the atmospherically corrected Level 2 A product. All processing was done with our own code and the Python packages eolearn (version 1.4.2) and sentinelhub (version 3.9.1).

According to the Sentinel-2 Annual Performance Report^64^, the absolute geometric accuracy of Sentinel-2 L2A data is better than 6 m, multi-temporal co-registration of the same or different satellites in the same repeat orbit is better than 5 m at 95% confidence, and multi-temporal co-registration in different repeat orbits is better than 5 m.

### Annotations

Many machine learning and deep learning studies in archaeological prospection use ALS visualisations with simple annotations that do not delineate the exact boundaries of objects. Such studies mostly use points and simple bounding boxes as annotations, which makes them primarily suitable for tasks dealing with object classification or object localisation (detection), rather than semantic segmentation. For segmentation purposes, the exact boundaries of an object are a prerequisite.

To create a dataset suitable for supervised segmentation, we delineated polygons for archaeological objects. Data annotation was done by a single person. The manual work took roughly 8 full working months and resulted in 9303 buildings and 2110 platforms annotated in the southern part (130 km^2^), and 95 aguadas annotated in the whole study area (220 km^2^) (Table 6). The platforms are apparently artificial, flat surfaces that stand out from the surrounding terrain, support other structures or most likely had this function, even if no buildings are currently visible. Buildings include various types of raised structures such as temple-pyramids, palace-like buildings, ball courts, single or multi-room houses and residential complexes. Aguadas are mostly clay-lined depressions capable of holding water through the dry season ^see also^^35^.

**Table 6:** The number of annotations.

	all annotated in the southern part of the study area	in data records	% of objects with less than 20% of their area on a data tile
aguada	51	51	0.0
building	9303	8275	4.6
platform	2110	1996	3.3

The perimeter polygon around a building or platform was drawn where the interpreter could define the boundary between artificial (modified) and natural terrain on VAT. Wherever possible, a single polygon represents a single instance, a single structure. However, because buildings are often closely connected architecturally or due to collapsed material, a precise boundary between structures is sometimes difficult to determine. As a result, a single polygon often contains more than one building. Polygons of buildings and platforms regularly overlap, but there are also many examples of platforms without buildings and of buildings that are not on a platform.

We used local dominance (LD) visualisation to complement the VAT for annotating aguadas and larger water reservoirs. LD is particularly suitable for depicting their slightly raised embankments.

All polygons of the same type (building, platform, or aguada) were saved as separate vector layers. The annotations were revised and curated by an expert archaeologist with deep local knowledge of the area. In very ambiguous cases where it was difficult to determine whether a formation is natural or anthropogenic, e.g. whether an object is a small, eroded platform or a naturally levelled terrain, consensus was reached within a panel of experts. We discussed the issues individually or agreed on a new rule if there were many similar examples.

The modified landscape contains many other types of anthropogenic structures such as terraces, quarries, walls, sacbeob (raised paved roads), chultunob (underground storage chambers), channels, rock piles etc. To save time, we did not annotate these initially, as we were primarily interested in the number and cumulative volume of buildings and the volume of available water to determine the number of people living in the area and the labour required to construct the structures in question. Because the study did not prioritize agricultural aspects, areas with channels in bajos and of terraces were delineated later and this data is not included in the data records. Other smaller and less pronounced anthropogenic structures, such as short walls, are also numerous and often eroded, making them harder to distinguish from natural formations. With the unlabelled data now made available, future researchers can annotate these existing records for further projects dealing with ancient Maya land use.

### Binary masks

Vector polygons representing each object class were rasterised to create a binary segmentation mask for that class. The rasterisation was done using the Geospatial Data Abstraction Library (GDAL). The masks were converted to TIFF files, and serve as labelled data for training, validation, and testing.

### Tiling

The fully annotated area of about 130 km^2^ was split into tiles of 240 × 240 meters. Each data record consists of tiles with multiple layers, except for the CHM which has only one layer (Fig. 5):

- three binary segmentation masks (one each for building, platform and aguada class),
- the ALS visualisations tile with three layers,
- the canopy height model,
- the Sentinel-1 image tile with multiple layers, and
- the Sentinel-2 image tile with multiple layers.

In accordance with the original DEM resolution of 0.5 m, visualisations and binary mask tiles each have a size of 480 × 480 pixels, the CHM has a size of 240 × 240 pixels, while the Sentinel-1 and 2 data have 24 × 24 pixels. The neighbouring tiles do not touch or overlap, but are separated by a buffer of 20 meters. The geographic location of tiles was chosen to match the 10 m grid of Sentinel-2 data.

## Data Records

**Fig. 6:**
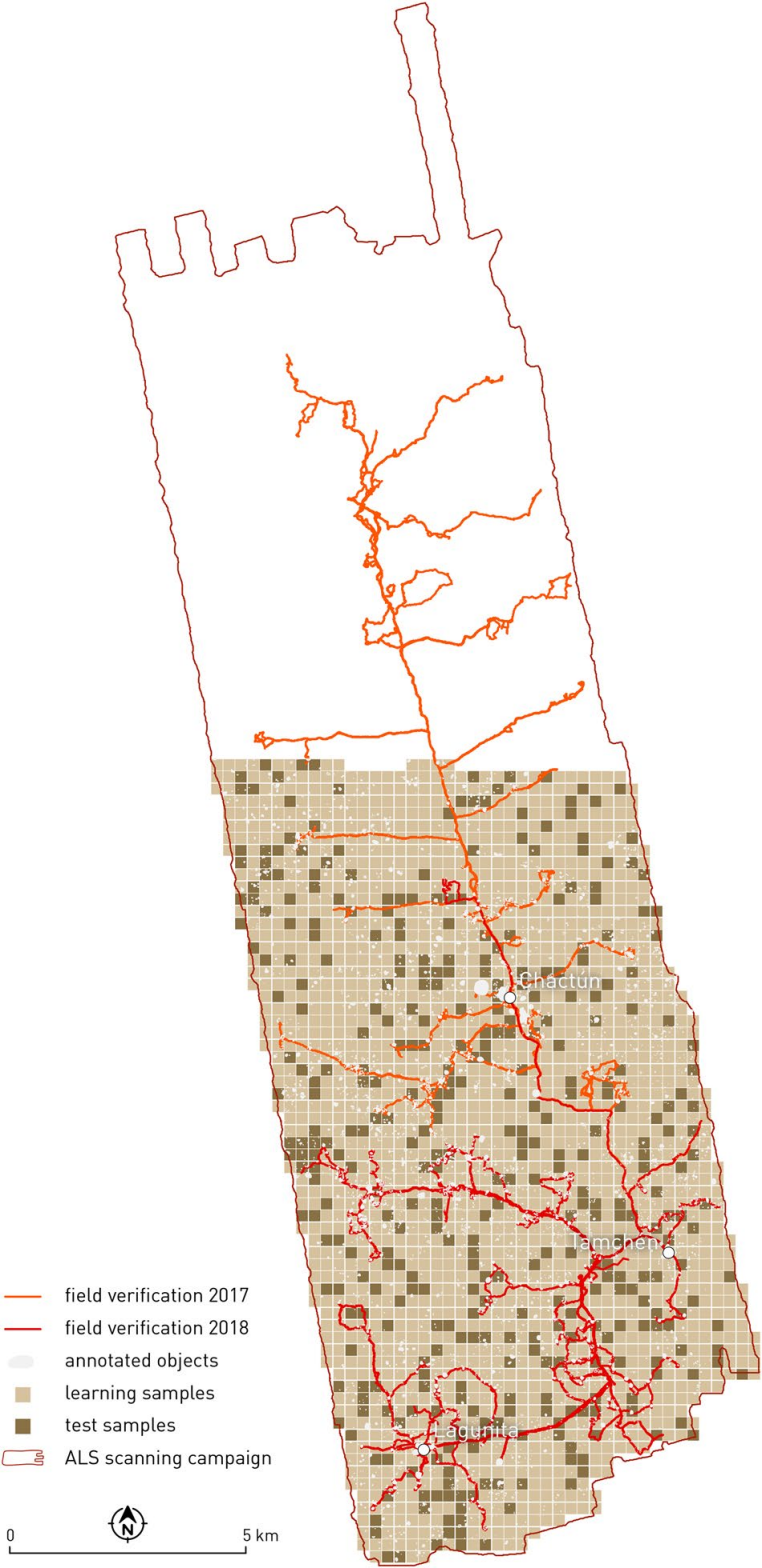
Learning (train and validation) and test tiles with ground tracks of two field verification campaigns.

Tiled binary masks, ALS visualisations, Sentinel-1, and Sentinel-2 satellite data are archived in Figshare online repository^65^. The dataset contains 2094 data records with an object in at least one of the segmentation masks. The randomly selected set of 1765 data records (tiles 0–1764) was initially published for the participants of the *Discover the mysteries of the Maya* online challenge that we organised in the framework of the European Conference on Machine Learning and Principles and Practice of Knowledge Discovery in Databases (ECML PKDD 2021)^48,49^, while the remaining 329 (tiles 1765–2093) were withheld for testing the submitted deep learning models (Fig. 6). The file format for all tiles is uncompressed TIFF. Geolocation data was intentionally omitted to avoid revealing the exact location of the archaeological remains. Tiles were randomly numbered to prevent reconstruction of the entire study area. An example of a data record with details of each tile can be found in Fig. 5 and in Tables 7, 8.

**Table 7:** Properties of a single data record (e.g. 1469).

name	bit depth	range	size	bands	pixel size	file size
tile_1469_mask_aguada.tif	8-bit	0, 255	480 px × 480 px	1	0.5 m	231 kB
tile_1469_mask_building.tif	8-bit	0, 255	480 px × 480 px	1	0.5 m	231 kB
tile_1469_mask_platform.tif	8-bit	0, 255	480 px × 480 px	1	0.5 m	231 kB
tile_1469_lidar.tif	8-bit	0–255	480 px × 480 px	3	0.5 m	692 kB
tile_1469_CHM.tiff	32-bit	0–30	240 px × 240 px	1	1 m	231 kB
tile_1469_S1.tiff	32-bit	0–1	24 px × 24 px	120	10 m	278 kB
tile_1469_S2.tiff	32-bit	0–1	24 px × 24 px	221	10 m	511 kB

**Table 8:** The structure of Sentinel-1 and Sentinel-2 data tiles.

Sentinel-1		Sentinel-2	
TIFF band	content description	TIFF band	content description
1	2017_ASC_VV_mean	1	2020-04-14_B01
2	2017_ASC_VV_median	2	2020-04-14_B02
3	2017_ASC_VV_std	3	2020-04-14_B03
4	2017_ASC_VV_var	4	2020-04-14_B04
5	2017_ASC_VV_p5	5	2020-04-14_B05
6	2017_ASC_VV_p95	6	2020-04-14_B06
7	2017_ASC_VH_mean	7	2020-04-14_B07
…	…	8	2020-04-14_B08
13	2017_DES_VV_mean	9	2020-04-14_B8A
…	…	10	2020-04-14_B09
19	2017_DES_VH_mean	11	2020-04-14_B11
…	…	12	2020-04-14_B12
25	2018_ASC_VV_mean	13	2020-04-14_CLM
…	…	14	2017-02-24_B01
120	2017-2020_DES_VH_p95	…	…
		221	2017-01-25_CLM

The filename structure for each data record is *tile_<consecutive-number>_<data-source>.tif*, where the data source can specify a mask, ALS visualisations (lidar), CHM, or Sentinel data (S1 or S2). The sequential number is a unique identifier of a data record; all files with the same sequential number represent the same geographical area, but differ in the number of pixels (480 × 480 pixels, 240 × 240 pixels or 24 × 24 pixels) and bit depth (8-bit integer or 32-bit float) (Table 7).

Each Sentinel-1 tile consists of 120 bands (5 periods × 24 bands) sorted as follows (Fig. 5 and Table 8):

- data are first ordered by year, with the entire period last (2017, 2018, 2019, 2020, 2017–2020), they are then
- ordered by the orbit (ascending – ASC, descending – DES),
- ordered by the corresponding polarization (VV, VH), and finally
- ordered by the calculated statistics (mean, median, standard deviation – std, coefficient of variance – var, 5^th^ percentile – p5, 95^th^ percentile – p95).

Each Sentinel-2 tile consists of 221 bands (17 dates × 13 bands), ordered by acquisition date (from newest to oldest), the corresponding spectral bands, and the associated cloud mask (Fig. 5 and Table 8).

A single data record is 2.35 Mb in size, while the total size by type is as follows: 1,449 MB for masks, 1,449 MB for ALS visualisations, 484 MB for CHM, 581 MB for Sentinel-1, and 1,070 MB for Sentnel-2 (a total of 5,033 MB). The repository stores ZIP compressed data, compiled by type (masks, lidar, CHM, S1, S2). The total size of the compressed files is 2,214 MB.

## Technical Validation

Because of the wide variety of data used to create the data records, we have described the process for obtaining the best possible input for each of the sources in the relevant section of the Methods chapter. The object boundaries resulting from the interpretation of the ALS data serve as ground truth. Based on the 10 m buffer from ground tracks from the 2017 and 2018 field verification campaigns, we verified 33.3% of aguadas, 22.4% of buildings, and 24.2% of platforms in the field (Fig. 6). Given the extreme difficulty of fieldwork in the remote and densely vegetated area, these are very high numbers. We did not record the errors systematically and cannot give exact frequencies for overlooked objects. However, the quality of our ALS data and the nature of the archaeological structures surveyed suggest that the number of structures we may have overlooked or mislabelled is likely to be very small. The experience of archaeologists working in the Neotropics shows that interpretations derived from ALS data are very reliable and that field verification can be less consistent over larger areas as conditions make efficient investigation impossible.

## Data Availability

ALS visualisations were calculated with Relief Visualization Toolbox (version 2.2.1), available at https://github.com/EarthObservation/RVT_py. The code for creating the satellite data records from Sentinel-1 and Sentinel-2 is available at https://github.com/EarthObservation/Sentinel-S1-S2-ML-patches-workflow.

## References

[1] Chase AF, Reese-Taylor K, Fernandez-Diaz JC, Chase DZ (2016). Progression and Issues in the Mesoamerican Geospatial Revolution: An Introduction. Advances in Archaeological Practice.

[2] Beach T (2015). Ancient Maya impacts on the Earth’s surface: An Early Anthropocene analog?. Quaternary Science Reviews.

[3] Canuto MA (2018). Ancient lowland Maya complexity as revealed by airborne laser scanning of northern Guatemala. Science.

[4] Chase AF (2011). Airborne LiDAR, archaeology, and the ancient Maya landscape at Caracol, Belize. Journal of Archaeological Science.

[5] Chase AF (2014). The Use of LiDAR in Understanding the Ancient Maya Landscape: Caracol and Western Belize. Advances in Archaeological Practice.

[6] Ebert CE, Hoggarth JA, Awe JJ (2016). Integrating Quantitative Lidar Analysis and Settlement Survey in the Belize River Valley. Advances in Archaeological Practice.

[7] Fisher CT, Cohen AS, Fernandez-Diaz JC, Leisz SJ (2017). The application of airborne mapping LiDAR for the documentation of ancient cities and regions in tropical regions. Quaternary International.

[8] Beach T (2019). Ancient Maya wetland fields revealed under tropical forest canopy from laser scanning and multiproxy evidence. PNAS.

[9] Dunning NP (2019). Margin for error: Anthropogenic geomorphology of Bajo edges in the Maya Lowlands. Geomorphology.

[10] Stanton TW (2020). ‘Structure’ density, area, and volume as complementary tools to understand Maya Settlement: An analysis of lidar data along the great road between Coba and Yaxuna. Journal of Archaeological Science: Reports.

[11] Šprajc I (2021). Ancient Maya water management, agriculture, and society in the area of Chactún, Campeche, Mexico. Journal of Anthropological Archaeology.

[12] Kokalj, Ž. & Hesse, R. *Airborne laser scanning raster data visualization: A guide to good practice*. (Založba ZRC, 2017).

[13] Kokalj Ž, Somrak M (2019). Why Not a Single Image? Combining Visualizations to Facilitate Fieldwork and On-Screen Mapping. Remote Sensing.

[14] Devereux BJ, Amable GS, Crow P (2008). Visualisation of LiDAR terrain models for archaeological feature detection. Antiquity.

[15] Horn SW, Ford A (2019). Beyond the magic wand: methodological developments and results from integrated Lidar survey at the ancient Maya Center El Pilar. STAR: Science & Technology of Archaeological Research.

[16] Hutson SR (2015). Adapting LiDAR data for regional variation in the tropics: A case study from the Northern Maya Lowlands. Journal of Archaeological Science: Reports.

[17] Inomata T (2020). Monumental architecture at Aguada Fénix and the rise of Maya civilization. Nature.

[18] von Schwerin J (2016). Airborne LiDAR acquisition, post-processing and accuracy-checking for a 3D WebGIS of Copan, Honduras. Journal of Archaeological Science: Reports.

[19] Chase, A. F. & Chase, D. Z. Detection of Maya Ruins by LiDAR: Applications, Case Study, and Issues. in *Sensing the Past: From artifact to historical site* (eds. Masini, N. & Soldovieri, F.) 455–468, 10.1007/978-3-319-50518-3_22 (Springer International Publishing, 2017).

[20] Hansen, R. D. *et al*. Developmental Dynamics, Energetics, and Complex Economic Interactions of the Early Maya of the Mirador-Calakmul Basin, Guatemala, and Campeche, Mexico. in *Pathways to Complexity* (eds. Brown, M. K. & Bey, G. J.) 147–194, 10.2307/j.ctvx075hx.12 (University Press of Florida, 2018).

[21] Inomata T (2017). Archaeological Application of Airborne LiDAR with Object-Based Vegetation Classification and Visualization Techniques at the Lowland Maya Site of Ceibal, Guatemala. Remote Sens..

[22] Jantz P, Goetz S, Laporte N (2014). Carbon stock corridors to mitigate climate change and promote biodiversity in the tropics. Nature Climate Change.

[23] Ruhl T, Dunning NP, Carr C (2018). Lidar reveals possible network of ancient Maya marketplaces in southwestern Campeche, Mexico. Mexicon.

[24] Banaszek Ł, Cowley DC, Middleton M (2018). Towards National Archaeological Mapping. Assessing Source Data and Methodology - A Case Study from Scotland. Geosciences.

[25] Verschoof-van der Vaart WB, Lambers K (2019). Learning to Look at LiDAR: The Use of R-CNN in the Automated Detection of Archaeological Objects in LiDAR Data from the Netherlands. Journal of Computer Applications in Archaeology.

[26] Bundzel M (2020). Semantic Segmentation of Airborne LiDAR Data in Maya Archaeology. Remote Sensing.

[27] Neupane B, Horanont T, Aryal J (2021). Deep Learning-Based Semantic Segmentation of Urban Features in Satellite Images: A Review and Meta-Analysis. Remote Sensing.

[28] Somrak M, Džeroski S, Kokalj Ž (2020). Learning to classify structures in ALS-derived visualizations of ancient Maya settlements with CNN. Remote Sensing.

[29] Esteva A (2017). Dermatologist-level classification of skin cancer with deep neural networks. Nature.

[30] Tj, B. *et al*. Deep neural networks are superior to dermatologists in melanoma image classification. *European journal of cancer (Oxford, England: 1990)* **119**, (2019). 10.1016/j.ejca.2019.05.023 31401469

[31] Rc, M. *et al*. Systematic outperformance of 112 dermatologists in multiclass skin cancer image classification by convolutional neural networks. *European journal of cancer (Oxford, England: 1990)* **119** (2019). 10.1016/j.ejca.2019.06.013 31419752

[32] Eskreis-Winkler S (2022). Breast MRI Background Parenchymal Enhancement Categorization Using Deep Learning: Outperforming the Radiologist. Journal of Magnetic Resonance Imaging.

[33] Pei L, Vidyaratne L, Rahman MM, Iftekharuddin KM (2020). Context aware deep learning for brain tumor segmentation, subtype classification, and survival prediction using radiology images. Sci Rep.

[34] Hirsch, L. *et al*. Radiologist-Level Performance by Using Deep Learning for Segmentation of Breast Cancers on MRI Scans. *Radiology: Artificial Intelligence*, 10.1148/ryai.200231 (2021). 10.1148/ryai.200231 PMC8823456 35146431

[35] Šprajc I (2022). Archaeological landscape, settlement dynamics, and sociopolitical organization in the Chactún area of the central Maya Lowlands. PLOS ONE.

[36] Caspari G, Crespo P (2019). Convolutional neural networks for archaeological site detection – Finding “princely” tombs. Journal of Archaeological Science.

[37] Bonhage A (2021). A modified Mask region-based convolutional neural network approach for the automated detection of archaeological sites on high-resolution light detection and ranging-derived digital elevation models in the North German Lowland. Archaeological Prospection.

[38] Davis DS, Caspari G, Lipo CP, Sanger MC (2021). Deep learning reveals extent of Archaic Native American shell-ring building practices. Journal of Archaeological Science.

[39] Davis, D. S. & Lundin, J. Locating Charcoal Production Sites in Sweden Using LiDAR, Hydrological Algorithms, and Deep Learning. *Remote Sensing* **13** (2021).

[40] Verschoof-van der Vaart, W. B. & Lambers, K. Applying automated object detection in archaeological practice: A case study from the southern Netherlands. *Archaeological Prospection* 1–17, 10.1002/arp.1833 (2021).

[41] Verschoof-van der Vaart WB, Landauer J (2021). Using CarcassonNet to automatically detect and trace hollow roads in LiDAR data from the Netherlands. Journal of Cultural Heritage.

[42] Kazimi B, Thiemann F, Sester M (2019). Semantic Segmentation of Manmade Landscape Structures in Digital Terrain Models. ISPRS Annals of Photogrammetry, Remote Sensing and Spatial Information Sciences.

[43] Soroush M, Mehrtash A, Khazraee E, Ur JA (2020). Deep Learning in Archaeological Remote Sensing: Automated Qanat Detection in the Kurdistan Region of Iraq. Remote Sensing.

[44] Guyot A, Lennon M, Lorho T, Hubert-Moy L (2021). Combined Detection and Segmentation of Archeological Structures from LiDAR Data Using a Deep Learning Approach. Journal of Computer Applications in Archaeology.

[45] Banasiak PZ (2022). Semantic Segmentation (U-Net) of Archaeological Features in Airborne Laser Scanning—Example of the Białowieża Forest. Remote Sensing.

[46] Küçükdemirci M, Landeschi G, Ohlsson M, Dell’Unto N (2023). Investigating ancient agricultural field systems in Sweden from airborne LIDAR data by using convolutional neural network. Archaeological Prospection.

[47] Kazimi, B., Thiemann, F. & Sester, M. Object Instance Segmentation in Digital Terrain Models. in *Computer Analysis of Images and Patterns* (eds. Vento, M. & Percannella, G.) 488–495, 10.1007/978-3-030-29891-3_43 (Springer International Publishing, 2019).

[48] Simidjievski, N. *et al*. Discover the mysteries of the Maya: ECML PKDD 2021 - Discovery Challenge. (2021).

[49] *Discover the mysteries of the Maya. Selected contributions from the machine learning challenge & the discveory challenge workshop, ECML PKDD 2021*. (Jožef Stefan Institute, 2022).

[50] Dunning, N. P. & Beach, T. Farms and Forests: Spatial and Temporal Perspectives on Ancient Maya Landscapes. in *Landscapes and Societies: Selected Cases* (eds. Martini, I. P. & Chesworth, W.) 369–389, 10.1007/978-90-481-9413-1_23 (Springer Netherlands, 2011).

[51] Beach T (2018). Stability and instability on Maya Lowlands tropical hillslope soils. Geomorphology.

[52] Šprajc, I. Introducción. in *Exploraciones arqueológicas en Chactún, Campeche*, México (ed. Šprajc, I.) 1–3 (Založba ZRC, 2015).

[53] Šprajc, I., Flores Esquivel, A. & Marsetič, A. Descripción del sitio. in *Exploraciones arqueológicas en Chactún, Campeche*, México (ed. Šprajc, I.) 5–24 (Založba ZRC, 2015).

[54] Šprajc I (2015). Chactún, Tamchén y Lagunita: primeras incursiones arqueológicas a una región ignota. Arqueología Mexicana.

[55] *Archaeological Reconnaissance in Eastern Campeche, Mexico: Chactun, Tamchen, and Lagunita*. (Tulane University, 2021).

[56] Fernandez-Diaz JC, Carter WE, Shrestha RL, Glennie CL (2014). Now You See It… Now You Don’t: Understanding Airborne Mapping LiDAR Collection and Data Product Generation for Archaeological Research in Mesoamerica. Remote Sens..

[57] Fernandez-Diaz JC (2016). Capability Assessment and Performance Metrics for the Titan Multispectral Mapping Lidar. Remote Sens..

[58] Axelsson, P. DEM Generation from Laser Scanner Data Using Adaptive TIN Models. in *International Archives of the Photogrammetry, Remote Sensing and Spatial Information Sciences - ISPRS Archives* vol. 33, 110–117 (International Society for Photogrammetry and Remote Sensing, 2000).

[59] Kokalj, Ž. & Oštir, K. Lidar data visualization and processing. in *The Encyclopedia of Archaeological Sciences* 1–6, 10.1002/9781119188230.saseas0347 (John Wiley & Sons, Ltd, 2018).

[60] Khosravipour A, Skidmore AK, Isenburg M (2016). Generating spike-free digital surface models using LiDAR raw point clouds: A new approach for forestry applications. International Journal of Applied Earth Observation and Geoinformation.

[61] Modified Copernicus Sentinel data (2017–2020) (2023). Sentinel Hub.

[62] Sentinel-1 Mission Performance Centre. *S-1 Annual Performance Report for 2022*. 117, https://sentinel.esa.int/documents/247904/4889382/DI-MPC-APR-0588-1-2-track-Annual+Performance+Report+2022.pdf/a683c9d2-06c2-9143-b456-4a9c8e30e449?t=1678771841042 (2023).

[63] Aleksandrov, M. *et al*. Sentinel Hub’s cloud detector for Sentinel-2 imagery. (2020).

[64] Sentinel-2 MSI Expert Support Laboratory team. *Sentinel-2 Annual Performance Report – Year 2022*. 132, https://sentinels.copernicus.eu/documents/247904/4893455/OMPC.CS.APR.001+-+i1r0+-+S2+MSI+Annual+Performance+Report+2022.pdf (2023).

[65] Kokalj Ž (2023). figshare.

[66] European Space Agency. *Sentinel-2 Spectral Response Functions (S2-SRF)*. 5, https://sentinels.copernicus.eu/documents/247904/685211/S2-SRF_COPE-GSEG-EOPG-TN-15-0007_3.1.xlsx (2022).

